# Patient-reported outcomes following treatment with the human GLP-1 analogue liraglutide or glimepiride in monotherapy: results from a randomized controlled trial in patients with type 2 diabetes

**DOI:** 10.1111/j.1463-1326.2010.01196.x

**Published:** 2010-07

**Authors:** B W Bode, M A Testa, M Magwire, P M Hale, M Hammer, L Blonde, A Garber

**Affiliations:** 1 Atlanta Diabetes AssociatesAtlanta, GA, USA; 2 Department of Biostatistics, Harvard School of Public HealthBoston, MA, USA; 3 Shawnee Mission Medical CenterMerriam, KS, USA; 4 Novo Nordisk IncPrinceton, NJ, USA; 5 Novo Nordisk A/SBagsvaerd, Denmark; 6 Department of Endocrinology, Diabetes and Metabolic Diseases, Ochsner Medical CenterNew Orleans, LA, USA; 7 Baylor College of MedicineHouston, TX, USA

**Keywords:** GLP-1, new treatments, quality of life, type 2 diabetes, weight issues

## Abstract

**Aim:** As weight gain and hypoglycaemia associated with glimepiride therapy can negatively impact weight perceptions, psychological well-being and overall quality of life in type 2 diabetes, we investigated whether liraglutide treatment could improve these factors.

**Methods:** Seven hundred and thirty-two patients with type 2 diabetes completed a 77-item questionnaire during a randomized, 52-week, double-blind study with liraglutide 1.2 mg (*n* = 245) or 1.8 mg (*n* = 242) compared with glimepiride 8 mg (*n* = 245).

**Results:** Mean (SE) decreases in glycated haemoglobin levels were greater with liraglutide 1.2 mg [−0.84 (0.08)%] and 1.8 mg [−1.14 (0.08)%] than glimepiride [−0.51 (0.08)%; p = 0.0014 and p < 0.0001, respectively]. Patients gained weight on glimepiride [mean (SE), 1.12 (0.27) kg] but lost weight on liraglutide [1.2 mg: −2.05 (0.28) kg; 1.8 mg: −2.45 (0.28) kg; both p < 0.0001]. Patient weight assessment was more favourable with liraglutide 1.8 mg [mean (SE) score: 40.0 (2.0)] than glimepiride [48.7 (2.0); p = 0.002], and liraglutide 1.8 mg patients were 52% less likely to feel overweight [odds ratio (OR) 0.48; 95% confidence interval (CI): 0.331–0.696]. Mean (SE) weight concerns were less with liraglutide [1.2 mg: 30.0 (1.2); 1.8 mg: 32.8 (1.2)] than glimepiride [38.8 (1.2); p < 0.0001 and p < 0.001, respectively], with liraglutide groups 45% less likely to report weight concern (OR 0.55, 95% CI: 0.41–0.73). Mean (SE) mental and emotional health and general perceived health improved more with liraglutide 1.8 mg [476.1 (2.8) and 444.2 (3.2), respectively] than glimepiride [466.3 (2.8) and 434.5 (3.2), respectively; p = 0.012 and p = 0.033, respectively].

**Conclusions:** Improved glycaemic control and decreased weight with liraglutide 1.8 mg vs. glimepiride can improve psychological and emotional well-being and health perceptions by reducing anxiety and worry associated with weight gain.

## Introduction

Although insulin therapy has been shown to improve the health outcomes of patients with type 2 diabetes, its initiation and intensification are commonly postponed because of concerns over the burden of daily injections, regimen complexity and the often attendant hypoglycaemia and weight gain [1,2]. Even though oral antihyperglycaemic agents are often favoured as initial therapies because their regimens may be less burdensome, they are also not without side effects, including hypoglycaemia and/or weight gain (sulphonylureas, meglitinides, thiazolidinediones), and gastrointestinal symptoms (biguanides, alpha-glucosidase inhibitors [3,4]). The impact of such side effects on patient-centred outcomes, such as body image, weight and health perceptions, psychological well-being and cognitive impairment, must be factored into treatment decisions. The importance of weight gain as a side effect of treatment has been given a lower priority compared with hypoglycaemia and gastrointestinal problems. However, it is a particular concern in this patient population and is increasingly reflected in treatment algorithms [3]. The most recent consensus statement for glycaemic control in type 2 diabetes issued by the American Association of Clinical Endocrinologists (AACE) and American College of Endocrinology (ACE) pointed out that the benefits of GLP-1 agonists, whereby approximately 30% of patients experience considerable weight loss, might in fact supersede transitory gastrointestinal side effects and the inconvenience of twice-daily injections [5]. Over 80% of patients with type 2 diabetes are already either overweight or obese [6], and thus weight gain is both physically and psychologically undesirable. It may also be a barrier to the continuation or intensification of many antidiabetic therapies [7]. Tailoring diabetes treatments to address patients' individual pathophysiology, while balancing the risk of hypoglycaemia and weight gain, is therefore a significant challenge.

Evidence has shown that the health-related quality of life (HRQoL) of people with type 2 diabetes is compromised compared with general population norms, especially for physical functioning and well-being [8]. A number of factors have been implicated, including poor glycaemic control [9], obesity [10,11], treatment side effects (e.g. hypoglycaemia [12]) and especially the presence of diabetes complications [8,13]. The complexity of diabetes regimens might also impact on HRQoL [8,14], resulting in reduced adherence to therapy [15] and, as a consequence, reduced therapeutic effectiveness.

Liraglutide is a once-daily analogue of human glucagon-like peptide-1 (GLP-1), and the molecule shares 97% of the amino acid sequence of native GLP-1. The efficacy and safety of liraglutide treatment has been compared with those of standard treatments across the continuum of care in type 2 diabetes in a comprehensive phase 3a trial programme [Liraglutide Effect and Action in Diabetes (LEAD) trial programme [16–21]]. The results of the LEAD-3 liraglutide monotherapy trial have previously been reported [18]. In this study of patients previously treated with diet and exercise or with half-maximal doses or less of oral antidiabetic (OAD) monotherapy, liraglutide monotherapy was associated with greater reductions in glycated haemoglobin (HbA1C) levels, weight, systolic blood pressure and with less hypoglycaemia than glimepiride monotherapy. Here, we report the impact of liraglutide and glimepiride monotherapies on patients' self-reported perceptions of body image, weight and weight concern, psychological well-being and distress, cognitive functioning and health using data from the LEAD-3 trial.

## Methods

### Overview of the LEAD-3 Trial

Methodological details and clinical results for the LEAD-3 trial have been published elsewhere [18] (Clinical trial no.: NCT00294723, registered at ClinicalTrials.gov). In this randomized, double-blind, double-dummy, parallel-group study, participants were recruited from Mexico (12 sites) and the United States (126 sites), starting on 7 February 2006, with the last patient visit on 7 November 2007. In the main clinical trial, patients were stratified by baseline diabetes treatment (diet and exercise vs. OAD monotherapy) and randomly allocated to once-daily treatment with liraglutide 1.2 mg (*n* = 251) or 1.8 mg (*n* = 247), or glimepiride 8 mg (*n* = 248) for 52 weeks. The primary clinical endpoint was the change in HbA1C levels from baseline to 52 weeks. Secondary clinical endpoints included changes in fasting plasma glucose (FPG), 8-point self-monitored plasma glucose profiles, body weight, incidence of hypoglycaemia and assessment of patient-reported outcomes. Local institutional review boards approved the protocol and patients provided written informed consent before trial-related activities were initiated. The study was conducted in accordance with the Declaration of Helsinki.

### Patient-reported Outcomes

The patient-reported outcome assessments were performed as part of the LEAD-3 trial, with separate methodologies and statistical analysis plans. The type 2 diabetes modules were developed by the Rand Corporation and Phase V Technologies, Inc. [22,23] and have been previously validated against a large reference database (4571 subjects in 13 clinical studies conducted in 1987–2000). Furthermore, the questionnaires have been used in 15 clinical trials of type 2 diabetes [9,24,25].

The battery of scales included in the LEAD-3 trial comprised 77 self-administered questions. A description of the weight perception, body image and quality of life scales and items is given in Appendix 2. The questionnaire was completed at screening (week −3) for some patients, and for all patients at baseline (week 0), during (week 28) and at the end of treatment (week 52), and at study exit for patients withdrawing prematurely. All questionnaires and evaluations were processed and analysed independently by a centralized health outcomes laboratory (Phase V Technologies, Inc., Wellesley Hills, MA) using the Phase V® Outcomes Information System.

### Statistical Analyses

The changes in six patient-reported outcome measures during 52 weeks of liraglutide or glimepiride treatment were compared. Analyses were performed on patients allocated to treatment, who received at least one dose of treatment and completed a baseline questionnaire. The screening questionnaire, if available, was used in place of a missed baseline assessment. Baseline and post-baseline values were carried forward to replace missing post-baseline values. To test the validity of these imputations, confirmatory analyses were conducted in two subgroups: (i) omitting patients with only baseline values and (ii) only patients completing both baseline and week 52 assessments. The effect of liraglutide and glimepiride treatments on the patient-reported outcomes was examined using linear mixed models. All treatment effects were adjusted for baseline score and age; country and sex were also included if statistically significant in the model. Post-randomization measures at weeks 28 and 52 were tested for between-week and treatment effects. Baseline to week 28 and baseline to week 52 changes were not evaluated separately as the week-by-treatment interaction effects were not statistically significant. Statistically significant between-week effects were retained in the models. Ordinal and logistic regression was used to model treatment differences between the proportions of individuals within the various weight-image and weight-concern response categories. Associations among patient-reported outcomes changes and glycaemic control were evaluated by correlation and regression. To examine the multivariate relationships and potential causal pathways within and between the multiple measures of weight perception and concern and quality of life components, structural equation modelling with latent variables was undertaken [26].

## Results

### Patient Disposition and Baseline Characteristics

Of the 746 patients enrolled, 14 opted not to participate in the patient-reported outcomes assessments. All 732 remaining patients completed a baseline assessment (liraglutide 1.2 mg, *n* = 245; liraglutide 1.8 mg, *n* = 242; glimepiride, *n* = 245) and 87.0% (637/732) of these also had at least one post-baseline assessment. Baseline demographic characteristics were similar across treatment groups (Table 1). The group was well educated, which was reflected in family income, and few patients reported disability or handicap. From the weight assessment scale, a total of 79.4% of patients considered themselves to be overweight [somewhat overweight: 47.1% (344/730); very overweight: 32.3% (236/730)] and, from the weight concern scale, 71.8% were concerned or worried about their weight [a little concerned: 24.0% (166/692); somewhat worried: 23.3% (161/692); very worried: 18.1% (125/692); extremely worried: 6.4% (44/692)]. Clinical baseline characteristics for this study have been detailed earlier [18]: mean (SD) disease duration, 6.0 (5.5) years; weight, 92.6 kg (19.6); body mass index (BMI), 33.1 (5.8); HbA1C level, 8.2% (1.1%); 64% of patients had previously been treated with OAD monotherapy and the remaining patients with diet and exercise only. Although this clinical dataset included the 14 patients who did not participate in the patient-reported outcome assessments reported here, the non-participants were statistically comparable to the participants.

**Table 1:** Demographic characteristics at baseline.

	Liraglutide 1.2 mg OD (*n* = 245)	Liraglutide 1.8 mg OD (*n* = 242)	Glimepiride 8 mg OD (*n* = 245)
Age in years, mean (SD)	53.8 (10.8)	52.2 (10.8)	53.3 (10.9)
Men/women, number (%)	113 (46)/132 (54)	119 (49)/123 (51)	131 (53)/114 (47)
Ethnic origin, number (%)			
White	194 (79)	183 (76)	190 (78)
Black or African American	34 (14)	29 (12)	29 (12)
Asian or Pacific Islander	5 (2)	13 (5)	9 (4)
Other	12 (5)	17 (7)	17 (7)
Ethnicity, number (%)			
Hispanic/Latino	80 (33)	85 (35)	92 (38)
Other	165 (67)	157 (65)	153 (62)
Married, number (%)	162 (67)	164 (69)	154 (65)
Education, number (%)			
Up to 18 years of age	123 (51)	100 (43)	109 (46)
More than 18 years of age	118 (49)	135 (57)	129 (54)
Occupation, number (%)			
Paid employment	149 (63)	153 (68)	163 (70)
Retired	52 (22)	38 (17)	34 (15)
Student	2 (1)	2 (1)	1 (<1)
Other	35 (15)	33 (15)	36 (15)
Family income in US$/year, number (%)			
<25 000	95 (40)	81 (35)	80 (35)
26 000–60 000	86 (36)	90 (39)	89 (39)
>60 000	56 (24)	59 (26)	61 (27)
Disability or handicap, number (%)	29 (12.1)	18 (7.7)	19 (8.1)

OD, once daily; SD, standard deviation.

Only patients completing a baseline patient-reported outcomes questionnaire are represented in this table.

### Clinical Endpoints

The results of the clinical endpoints from the LEAD-3 study are detailed by Garber and colleagues [18] and in Table 2. In summary, improvements in glycaemic control were greater in the liraglutide groups than the glimepiride group and, in pairwise comparisons, greater with the higher dose than the lower liraglutide dose. Reductions in HbA1C and FPG levels were significantly greater for liraglutide 1.2 mg and 1.8 mg vs. glimepiride (HbA1C: p = 0.0014 and p < 0.0001, respectively; FPG: p = 0.027 and p < 0.0001, respectively), and for liraglutide 1.2 mg vs. liraglutide 1.8 mg (HbA1C: p = 0.0046; FPG: p = 0.0223). Postprandial plasma glucose values (calculated from the 8-point self-monitored plasma glucose profiles) decreased in all treatment groups; the difference was significant for liraglutide 1.8 mg vs. glimepiride (p = 0.0038). Previously treatment-naïve patients experienced larger mean reductions in HbA1C than patients switched from an OAD drug and, in each case, those randomized to liraglutide experienced greater mean reductions than those receiving glimepiride; HbA1C reductions were sustained for 52 weeks of the study [18]. The liraglutide groups lost weight whereas the glimepiride group gained weight; the differences in change in body weight from baseline were significantly different for each liraglutide group vs. the glimepiride group (both p < 0.0001). No major hypoglycaemic events were reported. The rate of minor hypoglycaemia was significantly lower for each liraglutide group than for the glimepiride group (both p < 0.0001).

**Table 2:** Clinical endpoints from the study and some data previously reported by Garber and colleagues [18].

	Mean (SE)			Mean differences between treatment group (95% CI)		
	Liraglutide 1.2 mg OD (*n* = 251)	Liraglutide 1.8 mg OD (*n* = 247)	Glimepiride 8 mg OD (*n* = 248)	Liraglutide 1.2 mg vs. liraglutide 1.8 mg	Glimepiride vs. liraglutide 1.2 mg	Glimepiride vs. liraglutide 1.8 mg
Weight, kg						
Baseline	92.5 (1.21)	92.8 (1.32)	93.4 (1.22)			
Week 52 LOCF	90.2 (1.22)	90.5 (1.31)	94.4 (1.23)			
Change from baseline	−2.05 (0.28)	−2.45 (0.28)	1.12 (0.27)	−4.06	−3.17	−3.52
				(−1.11 to 0.30)	(−3.87 to −2.47)†	(−4.28 to −2.87)†
HbA1c levels, %						
Baseline	8.3 (0.06)	8.3 (0.07)	8.4 (0.08)			
Week 52 LOCF	7.5 (0.09)	7.2 (0.08)	7.8 (0.08)			
Change from baseline	−0.84 (0.08)	−1.14 (0.08)	−0.51 (0.08)	−0.29%	−0.33%	−0.62%
				(−0.50 to −0.09)**	(−0.53 to −0.13)**	(−0.83 to −0.42)†
FPG, mmol/l						
Baseline	9.3 (0.17)	9.5 (0.17)	9.5 (0.17)			
Week 52 LOCF	8.7 (0.20)	8.3 (0.18)	9.3 (0.19)			
Change from baseline	−0.84 (0.19)	−1.42 (0.19)	−0.29 (0.19)	−0.58	−0.55	−1.13
				(−1.07 to −0.08)*	(−1.04 to −0.06)*	(−1.62 to −0.64)†
Postprandial						
plasma glucose‡, mmol/l						
Baseline	11.3 (0.15)	11.4 (0.16)	11.4 (0.17)			
Week 52 LOCF	9.7 (0.20)	9.3 (0.16)	10.0 (0.19)			
Change from baseline	−1.71 (0.19)	−2.08 (0.19)	−1.36 (0.18)	−0.37	−0.35	−0.72
				(−0.85 to 0.11)	(−0.83 to 0.14)	(−1.20 to −0.23)**
				Rate ratio estimate (95% CI)		
Rate of minor hypoglycaemia,	0.30	0.25	1.96	0.62	0.16	0.10
events/patient				(0.29 to 1.30)	(0.08 to 0.32)†	(0.05 to 0.20)†
year at week 52						

FPG, fasting plasma glucose; CI, confidence interval; HbA1C, glycated haemoglobin; LOCF, last observation carried forward; OD, once daily; SE, standard error.

* p < 0.05.

** p < 0.01.

† p < 0.0001.

‡ Calculated from the 8-point self-monitored plasma glucose profiles.

### Effect of Treatment on Patient-reported Outcomes

As shown in Table 3, the mean scores of all patient-reported follow-up measures were estimated for the three treatment groups adjusting for baseline score and age (53.1 years). Both measures of weight perception (weight assessment and weight concern) were more favourable for liraglutide than glimepiride. Baseline-adjusted mean weight assessment compared with the reference point ‘my weight is just right' was significantly more favourable (i.e. shifted from more overweight to less overweight) for the liraglutide 1.8 mg group than the glimepiride group (p = 0.002; Table 3).

**Table 3:** Effect of treatment on patient-reported outcomes.

		Mean (SE) during treatment				Contrasts	
Patient-reported outcomes scale	Baseline covariate score (*n* = 732)	Liraglutide 1.2 mg OD	Liraglutide 1.8 mg OD	Liraglutide 8 mg OD	Between- treatment F statistic p-value	Glimepiride vs. liraglutide 1.2 mg p-value§ (p-value)¶	Glimepiride vs. liraglutide 1.8 mg p-value§ (p-value)¶
Weight perception scales							
Weight assessment−	50.6	47.6 (2.0)	40.0 (2.0)**	48.7 (2.0)	0.003	0.693	0.002
Weight concern−	37.8	30.0 (1.2)‡	32.8 (1.2)†	38.8 (1.2)	<0.0001	<0.0001 (0.0001)	<0.001 (0.019)
Body image scales							
Body size evaluation−	32.8	27.9 (0.9)	27.4 (0.9)	29.7 (0.9)	0.189	0.167	0.085
Body appearance distress+	44.0	45.5 (0.6)	47.0 (0.6)	45.5 (0.6)	0.169	0.963	0.108
HRQoL scales							
Composite HRQoL+	450.4	453.8 (2.6)	462.3 (2.6)**	451.7 (2.6)	0.009	0.556	0.004
Mental and emotional health+	464.6	464.4 (2.7)	476.1 (2.8)*	466.3 (2.8)	0.005	0.631	0.012
Psychological well-being+	411.5	409.5 (3.1)	424.2 (3.1)**	412.5 (3.1)	0.002	0.504	0.008
General positive affect+	408.7	406.5 (3.2)	422.2 (3.2)*	411.7 (3.2)	0.002	0.255	0.022
Life satisfaction+	422.8	422.1 (3.5)	436.9 (3.6)	428.2 (3.6)	0.013	0.227	0.084
Emotional ties+	433.1	432.8 (6.6)	442.5 (6.7)*	418.6 (6.8)	0.042	0.134	0.012
Psychological distress+	496.6	497.7 (2.9)	507.3 (2.9)*	498.4 (2.9)	0.032	0.854	0.030
Anxiety+	472.3	473.8 (3.3)	482.7 (3.3)	474.0 (3.3)	0.090	0.960	0.061
Depression+	531.1	530.8 (3.4)	542.2 (3.4)	535.4 (3.4)	0.057	0.339	0.154
Behavioural and emotional control+	509.2	506.1 (3.4)	524.2 (3.4)‡	505.1 (3.4)	0.000	0.842	<0.0001
General perceived health+	430.3	441.4 (3.2)	444.2 (3.2)*	434.5 (3.2)	0.089	0.127	0.033
Vitality+	394.8	407.7 (4.0)	415.3 (4.0)	401.8 (4.0)	0.058	0.305	0.017
General health status+	432.6	451.9 (3.7)**	446.2 (3.7)	437.5 (3.7)	0.022	0.006	0.096
Composite cognitive functioning and performance+	4.03	4.03 (0.02)	4.08 (0.02)	4.05 (0.04)	0.202	0.525	0.262

SE, standard error; OD, once daily; HRQoL, health-related quality of life.

+ indicates that higher scores are better.

− indicates that lower scores are better.

* p < 0.05

** p < 0.01

† p < 0.001

‡ p < 0.0001 vs. glimepiride.

§ p-value for treatment differences using linear mixed models and all available visit data adjusted for baseline score and age (53.1 years).

¶ p-value for treatment differences using ordinal regression on last observation carried forward adjusted for baseline score, age, and sex. All p-values are nominal unadjusted for multiple comparisons.

Furthermore, weight concern decreased markedly in the liraglutide groups with mean scores significantly less than for glimepiride (liraglutide 1.2 mg, p < 0.0001; liraglutide 1.8 mg, p < 0.001). Logistic regression estimates indicated that individuals receiving liraglutide 1.8 mg were 52% less likely to report feeling either ‘somewhat’ or ‘very overweight' vs. ‘just right', ‘somewhat underweight' or ‘very overweight' during treatment than those receiving glimepiride [odds ratio (OR) 0.48, 95% confidence interval (CI): 0.331–0.696]. Also, patients receiving liraglutide 1.8 mg were 39% less likely to report, on the weight concern scale, being ‘somewhat worried’, ‘very worried’ or ‘extremely worried’ vs. ‘a little concerned’ or ‘not concerned at all’ about their weight during treatment than those receiving glimepiride (OR 0.608, 95% CI: 0.44–0.85 by logistic regression). Patients receiving liraglutide 1.2 mg reported a 50% lower likelihood of weight concern (using the dichotomous classification cited above) than those receiving glimepiride (OR 0.50, 95% CI: 0.36–0.70). The OR for both liraglutide doses compared for the dichotomous weight concern variable was 0.55 (95% CI: 0.41–0.73).

There were no statistically significant differences between liraglutide groups and the glimepiride group for the body image scales (body size evaluation and body appearance distress) or for any of the cognitive functioning and performance scales during treatment. Furthermore, the results of analyses were comparable and consistent when performed omitting baseline observations carried forward and when only patients with week 52 data were included.

As shown in Table 3, the HRQoL composite score improved more favourably with liraglutide 1.8 mg than with glimepiride (p = 0.004). These favourable improvements were seen in the composite scales of mental and emotional health, psychological well-being, psychological distress and general perceived health (all p < 0.05). The higher scores with liraglutide 1.8 mg for mental and emotional health reflected greater improvement in both domains of psychological well-being (which included subscales of general positive affect, life satisfaction and emotional ties) and psychological distress (which included subscales of anxiety, depression, behavioural and emotional control) than glimepiride. There were no significant differences for these scales between liraglutide 1.2 mg and glimepiride. There was, however, a significant difference between liraglutide 1.2 mg and glimepiride in general health status (p = 0.006); the difference being in favour of liraglutide 1.2 mg. The more favourable HRQoL scores for liraglutide 1.8 mg compared with glimepiride were mediated predominantly by greater improvements in the subscales of emotional and behavioural control (p < 0.0001), general positive affect (p = 0.022), emotional ties (p = 0.012), life satisfaction (p = 0.084), vitality (p = 0.017) and general health status (p = 0.096). Other subscales contributed more moderately to overall treatment differences. As with the weight assessment, the results of analyses were comparable and consistent when performed omitting baseline observations carried forward and when only patients with week 52 data were included.

### Associations Between Changes in Patient-reported Outcomes and Glycaemic Control

Correlation analyses using data pooled from all treatment groups confirmed that baseline-to-endpoint reductions in BMI were correlated with baseline-to-endpoint improvements in both weight assessment and weight concern (r = 0.19 and r = 0.25, respectively; p < 0.0001 in both cases), indicating that patients' reports were valid representations of actual weight losses. In addition, baseline-to-endpoint reductions in HbA1C corresponded to improvements in general perceived health (r = −0.142, p < 0.0001), cognitive functioning composite score (r = −0.11, p = 0.006) and cognitive performance (r = −0.11, p = 0.004). Correlations of change in HbA1C within treatment groups with change in patient-reported measures were strongest for liraglutide 1.8 mg: general perceived health, r = −0.19, p = 0.004; cognitive functioning composite score, r = −0.18, p = 0.008; and cognitive performance, r = −0.19, p = 0.004.

**Figure 1:**
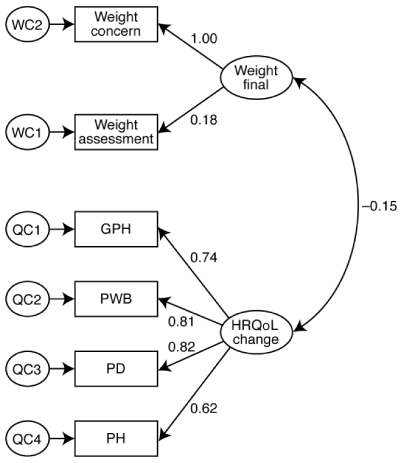
Structural equation model using data pooled from all treatment groups. The relative contributions of patient-reported outcomes (e.g. weight concern) to structural components (e.g. weight final) are detailed on the straight arrows. The correlation between structural components is detailed above the curved arrow for all groups combined; analyses within treatment groups gave estimates of −0.15, −0.22 and 0.03 for the liraglutide 1.2 mg, liraglutide 1.8 mg and glimepiride groups, respectively. GPH, general perceptions of health HRQoL, health-related quality of life; PD, psychological distress; PH, analogue perceived health; PWB, psychological well-being; WC, weight component; QC, quality of life component.

Two latent variables (weight perceptions and HRQoL) were constructed from linear combinations of the observed scales to represent treatment changes using a structural equation model fit to the baseline-to-endpoint change scores. The model represented a very good fit to the actual data (comparative fit index, 0.98; root mean square error of approximation, 0.055; PCLOSE, 0.322; Figure 1). The beta weights indicate the contribution of the specific scale on the latent variable with larger weights having greater influence. As shown here, the change in the weight perceptions latent variable was more heavily influenced by weight concern [standardized beta weight (*β*) = 1.00] than weight assessment (*β* = 0.18), indicating that the anxiety associated with weight gain was more influential than the perceived amount of weight gained during treatment. The *β* values for the scales associated with the HRQoL latent variable were relatively comparable, indicating similar contributions of each scale on HRQoL. The structural equation model indicated a statistically significant association between HRQoL and weight concern (*β* = −0.15, p < 0.001), supporting the link between decreased weight concern and improved HRQoL. The structural equation model for liraglutide 1.8 mg indicated that a decrease of 1 SD in weight concern was associated with a 22% SD unit improvement in the HRQoL latent variable (*β* = −0.22, p < 0.001).

## Discussion

The clinical assessments in the LEAD-3 trial showed that, compared with glimepiride, liraglutide monotherapy was associated with greater improvements in glycaemic control and a low incidence of hypoglycaemia over 52 weeks [18]. Moreover, liraglutide treatment was associated with weight loss rather than the weight gain seen with glimepiride. These previously reported findings provide the clinical context for the data reported here. In our study, patients' assessments of and concerns about their weight improved significantly more during treatment with liraglutide than with glimepiride. Furthermore, these more favourable treatment effects were greatest for the 1.8 mg dose of liraglutide. Taken together, patients in the liraglutide 1.8 mg and 1.2 mg dose groups were 45% less likely to report being ‘somewhat’ to ‘extremely concerned’ about their weight during treatment compared with before treatment. Moreover, those receiving 1.8 mg of liraglutide were 52% less likely to report feeling ‘somewhat’ or ‘very overweight' than those receiving glimepiride. These patient reports were consistent with the actual weight loss reported during the study. The absence of significant differences between treatment groups for the body image scales suggests that the body areas are not those of specific concern to this study population.

Compared with glimepiride, 1.8 mg liraglutide treatment was also associated with statistically significantly greater improvements in two domains of HRQoL: mental and emotional health, and general perceived health. As reported previously, some improvement is likely because of greater glycaemic control, which has been shown to increase vitality and general health functioning [9]. Although the treatment effects were in the same direction, the failure of the liraglutide 1.2 mg group to achieve statistical significance compared with glimepiride for the QoL scales could be because of the smaller decrease in glycaemic control in the 1.2 mg group compared with the 1.8 mg group.

However, the mental health improvements shown in this study for behavioural and emotional control, life satisfaction, emotional ties and general positive affect were most likely driven by a combination of greater glycaemic control, greater weight loss and perception of weight loss, and reduced concern about weight gain—all of which achieved statistical significance in the liraglutide 1.8 mg group. Emotional ties, for example, reflect how much of the time individuals feel that their love relationships are full and complete. The strong psychological impact of liraglutide 1.8 mg treatment was not solely the result of reductions in the symptoms of hyperglycaemia. The increased concern about weight gain and weight appearance for those receiving glimepiride translated into increased psychological distress and reduced well-being. Using structural equation modelling, the relationship between decreases in patients' perception of their weight and distress with weight gain and improvements in the overall composite score of the HRQoL measure was confirmed. Although it is not possible to determine the direction of this causal pathway, it is highly likely that the decrease in weight concern fuelled improvements in HRQoL.

This relationship between patients' concern about their weight during diabetes therapy and HRQoL has not previously been reported for patients with type 2 diabetes. The data in this study support clinical observations that weight loss and the absence of weight gain during liraglutide treatment result in a meaningful improvement in HRQoL, which is mediated at least in part by reduced concerns and distress about weight. This is an important finding, as weight control is a major issue in the management of type 2 diabetes. The dose-response effect, whereby the 1.8 mg dose of liraglutide showed a greater improvement compared with glimepiride than did the liraglutide 1.2 mg dose compared with glimepiride, supports a causal relationship between liraglutide treatment and improvements in HRQoL.

Over 80% of patients with diabetes are overweight and many present with hypertension, a major risk factor for coronary artery disease. This is exacerbated by many existing treatments, which improve glycaemic control at the expense of weight gain [3]. In addition, weight gain (or fear of weight gain) is a major negative influence on treatment adherence [15]. In the development of the AACE/ACE type 2 diabetes treatment algorithm, six goals were established as priorities in the selection of medications. One of these goals was ‘minimizing risk and magnitude of weight gain’. In light of this, a treatment that results in both weight loss and demonstrable changes in patients' concern about their weight which can positively impact QoL represents a major advance in the management of this disease, potentially facilitating improved adherence and glycaemic control.

Patients' concern and distress because of weight gain differentials during treatment were not simply psychosomatic manifestations as the data also showed that changes in BMI were correlated with changes in patients' perceptions and distress with weight. The data also confirmed that concerns and perceptions were significantly related to changes in an overall score reflecting mental and emotional health and general perceived health. The improvement in HbA1C levels during treatment was also found to be related to improvements in HRQoL. Associations between improved glycaemic control and aspects of HRQoL have been reported earlier, but they are not evident in all investigations [8,9,27]. Testa and Simonson suggested that the relationship between glycaemic control and HRQoL may sometimes be masked by the adverse effects of treatment-related hypoglycaemia on HRQoL [9]. As the rate of hypoglycaemia is very low with liraglutide, this may also be the reason that a glycaemic control–HRQoL relationship was detected in the present study.

Collectively, the findings from the LEAD-3 trial suggest that, through the complex interplay of clinical and patient-reported outcome benefits, liraglutide treatment has the potential to impact positively on patients' adherence to treatment, which is critical to the achievement of glycaemic targets. Recent data from the USA show that approximately 40% of patients with type 1 or type 2 diabetes have HbA1C levels in excess of the 7% treatment target [28], and thus remain at increased risk of various health complications. A treatment that results in improved self-management is likely to bring more of these patients under better glycaemic control.

Several possible limitations to the study should be considered. Firstly, post-baseline data were imputed for 95 patients (13.0%). However, the imputation method carried baseline and last observations forward, acting to reduce estimates of the treatment effect. As analytic models not using imputed data gave comparable results to those presented here, we are confident that discontinuation after randomization did not introduce bias. Secondly, although the nature and extent of the significant treatment results support the robustness of analyses, the pattern of changes during the year-long study could potentially have been more fully characterized by including more assessment points. Finally, we have not assessed the impact of liraglutide on other important patient-centred health measures, such as treatment satisfaction and adherence, which would be useful goals of future research.

In summary, the analyses from this large, active-controlled clinical trial of patients with type 2 diabetes show that liraglutide 1.8 mg treatment by once-daily injection is associated with significantly greater improvements in self-reported perceptions of body weight and measures of HRQoL than oral glimepiride treatment. What has also been shown here, and for the first time, is a significant association between decreasing concerns about weight and improvements in key domains of HRQoL. Given the influence of patient-centred factors on self-management behaviours in type 2 diabetes, these collective findings suggest liraglutide treatment may be a useful therapy for achieving glycaemic targets.

## Appendix 1. List of participating investigators

### LEAD-3 Study Group

Abbott L, Abelseth J, Alvarado-Ruiz R, Anjani D, Arakaki R, Arechavaleta-Granell M, Austin J, Bailey T, Barrera J, Bertolino J, Bettis R, Blonde L, Bode B, Bressler P, Brusco O, Camacho P, Carroll M, Cartas M, Cefalu W, Cheatham W, Chisolm O, Clarke D, Corbett B, Corder C, Cox M, Crockett S, Cullen E, DeHaven J, Downey H, Duckor S, East H, Farmer H, Farrell J, Feinglos M, Fishman N, Fusco F, García-Hernández P, Garber A, Gilbert J, Gilman R, Goldstein B, Gollapudi G, Gonzalez-Galvez G, Gonzalez-Villalpando C, Graf R, Greco S, Griffing G, Hartman I, Hassman D, Hodge R, Hoffman B, Hollander P, Huffman D, Hunt G, Jain R, Kaplan R, Kapoor A, Kawley A, Klein E, Landgarten S, Leichter S, Leslie H, Licata A, Linden D, Lipetz R, Lochner J, Magee M, Mar-Arevalo F, Mather K, McGill J, Mezitis N, Molitch M, Morales-Flores H, Morris L, Mudaliar S, Mulmed L, Myers L, O’Barr T, Olansky L, Ollins R, Olvera-Alvarez I, Palte S, Pearlstein R, Peterson G, Phillips L, Popeil L, Powers C, Pullman J, Race J, Ratcliff L, Reichman A, Reeves M, Reynolds L, Rios-Rodriguez E, Rivera-Colon L, Robinson M, Rodriguez-Pattzi H, Rosenstock J, Rothman J, Salinas-Gonzalez F, Sauque-Reyna L, Schumacher D, Schwartz S, Seidman B, Sharma S, Shelmet J, Shepherd M, Shomali M, Silver B, Smith T, Snell P, Snyder B, Sosa-Camas R, Sugimoto D, Sussman A, Tamayo R, Tamez-Perez H, Thigpen D, Tisovec R, Trevino M, Violante-Ortiz R, Wahl T, Warren M, Weinrib S, Weinstein R, Weinstock R, Weiss D, Williams R, Winer N, Witkin D, Wittmer B, Zieve F, Zisser H.

## Appendix 2. Quality of life, weight perceptions and body image scales

### Weight Perception Scales

**Weight assessment:** one-item, five-choice, bidirectional Likert scale reflecting the subject's perception of their weight that day. Question stem was: ‘*Considering your weight today, do you consider yourself to be…..*’; response choices were: (i) very underweight, (ii) somewhat underweight, (iii) my weight is just right, (iv) somewhat overweight, and (v) very overweight.

**Weight concern:** one-item, five-choice, unidirectional Likert scale assessing the subject's distress and concern associated with his or her weight. Question stem was: ‘*Thinking about your weight now and your weight before you started the study drugs you are now taking, how concerned are you about your weight now?’*; response choices were: (i) extremely worried and upset, (ii) very worried, (iii) somewhat worried and concerned, (iv) a little concerned, and (v) not concerned at all.

### Body Image Scales

Seven items for each scale (body size evaluation and body appearance distress) assessing the subject's perception of the relative size of seven different body parts [face, breasts, belly, dorsal fat (buffalo hump), legs, arms, buttocks] compared with self-anchored ‘healthy look’. The question stem was *‘Think about how you would look if you were “healthy”. Now consider your “current appearance”. Compared to your healthy look, please comment about how your feel regarding the following areas of your body*.’ Sample items for belly size are shown below:

### Body Appearance Distress

**Table:**

Body size evaluation codes	Body appearance distress codes
*Compared to my healthy look my current amount or size is*	
1 = A great deal less/very much smaller or thinner	1 = Extremely upsetting and distressing
2 = A lot less/much smaller or thinner	2 = Very upsetting and distressing
3 = Somewhat less/smaller or thinner	3 = Quite upsetting and distressing
4 = A little less/smaller or thinner	4 = A little upsetting
**5** = **About right**	**5** = **No feeling either way**
6 = A little more or bigger	6 = A little encouraging
7 = Somewhat more or bigger	7 = Quite encouraging
8 = A lot more or bigger	8 = Very encouraging
9 = A great deal more or bigger	9 = Extremely encouraging

### Health-related Quality of Life Scales

**Mental and emotional health:** 24 questions with subscales of anxiety, depression, and loss of behavioural and emotional control **(psychological distress)**, life satisfaction, general positive affect and emotional ties **(psychological well-being)**.

**General health perceptions:** 11 questions with subscales of sleep disturbance, vitality and general health status.

**Composite health-related quality of life:** mean of all subscales of mental and emotional health and all subscales of general health perceptions.

**Composite cognitive function and performance:** 15 questions assessing self-reported subscales of cognitive acuity, disorientation and detachment and six questions on self-rated cognitive performance.

**Analogue perceived health:** five questions: patients rated how they had been feeling during the past month on a scale of 1–10 for each of the following categories: (i) overall or in general, (ii) physically, (iii) emotionally, (iv) personal life, and (v) about job or work.
